# Overexpression of prostate specific membrane antigen by canine hemangiosarcoma cells provides opportunity for the molecular detection of disease burdens within hemorrhagic body cavity effusions

**DOI:** 10.1371/journal.pone.0210297

**Published:** 2019-01-02

**Authors:** Matthew Dowling, Jonathan Samuelson, Bahaa Fadl-Alla, Holly C. Pondenis, Mark Byrum, Anne M. Barger, Timothy M. Fan

**Affiliations:** 1 Department of Veterinary Clinical Medicine, University of Illinois, Urbana, IL, United States of America; 2 Department of Pathobiology, University of Illinois, Urbana, IL, United States of America; Colorado State University, UNITED STATES

## Abstract

**Background:**

Canine hemangiosarcoma (cHSA) is a highly metastatic mesenchymal cancer that disseminates by hematogenous and direct implantation routes. Therapies for cHSA are generally ineffective, in part due to advanced clinical disease stage at the time of diagnosis. The validation of conventional molecular methods for detecting novel biomarkers preferentially expressed by cHSA could lead to more timely diagnosis, earlier therapeutic interventions, and improved outcomes. In humans, prostate-specific membrane antigen (PSMA) is a transmembrane protein overexpressed by prostate carcinoma and tumor-associated endothelium of various solid cancer histologies. Importantly, the preferential overexpression of PSMA by certain cancers has been leveraged for the development of diagnostic molecular imaging reagents and targeted therapeutics. Recently, PSMA has been qualitatively demonstrated to be expressed in cHSA cell lines, however, quantitative PSMA expressions and the potential utility of PSMA transcript identification in biologic fluids to support the presence of microscopic cHSA burden has not been reported. Therefore, this study sought to characterize the differential quantitative expressions of PSMA between cHSA and non-malignant tissues, and to determine the potential diagnostic utility of PCR-generated PSMA amplicons as a surrogate of rare cHSA cells dwelling within peritoneal and pericardial cavities.

**Methods:**

Quantitative gene and protein expressions for PSMA were compared between one normal endothelial and six cHSA cell lines by RT-PCR, western blot analysis, and fluorescent microscopy. Additionally, gene and protein expressions of PSMA in normal canine tissues were characterized. Graded expressions of PSMA were determined in spontaneously-arising cHSA tumor samples and the feasibility of qualitative PCR as a molecular diagnostic to detect PSMA transcripts in whole blood from healthy dogs and hemorrhagic effusions from cHSA-bearing dogs were evaluated.

**Results:**

PSMA gene and protein expressions were elevated (up to 6-fold) in cHSA cells compared with non-malignant endothelium. By immunohistochemistry, protein expressions of PSMA were detectable in all cHSA tissue samples evaluated. As predicted by human protein atlas data, PSMA’s expression was comparably identified at substantial levels in select normal canine tissues including kidney, liver, and intestine. In young healthy pet dogs, PSMA amplicons could not be identified in circulating whole blood yet were detectable in hemorrhagic effusions collected from pet dogs with confirmed cHSA or PSMA-expressing cancer.

**Conclusions:**

PSMA is quantitatively overexpressed in cHSA compared to normal endothelium, but its protein expression is not restricted to only cHSA tumor tissues, as specific visceral organs also substantively express PSMA. Optimized qualitative PCR methods failed to amplify PSMA amplicons sufficiently for visible detection in circulating whole blood derived from healthy young dogs, yet PSMA transcripts were readily identifiable in hemorrhagic effusions collected from pet dogs with histologically confirmed cHSA or PSMA-expressing cancer. While preliminary, findings derived from a limited cohort of normal and diseased pet dogs provocatively raise the potential value of PSMA amplicon detection as an ancillary molecular diagnostic test for supporting the presence of microscopic cHSA disease burden within hemorrhagic body cavity effusions.

## Introduction

Molecular diagnostic tests employ the use of advanced technologies to detect DNA, RNA, proteins, or specific metabolites that are associated with certain pathologic conditions even when those diseases are below the limit of clinical detection. In oncology, molecular diagnostic testing has revolutionized cancer diagnosis and treatment in people, which has ultimately led to improved outcomes in a wide variety of solid and hematopoietic malignancies [1, 2]. Analogously in veterinary medicine, with the growing availability and diminished cost of molecular diagnostic platforms, there has been increased utilization of various methodologies for improving the diagnosis and treatment of common ailments including cancer that afflict companion animals [3]. In veterinary oncology, some notable examples of clinically impactful molecular diagnostics include flow cytometry, gene sequencing, and polymerase chain reaction (PCR)-based techniques, such as PCR for antigen receptor rearrangement (PARR) for lymphoid malignancies, c-Kit gene mutation detection for mast cell disease, BRAF gene mutation detection for urothelial carcinomas, and MDR1 gene mutation detection for guiding chemotherapy dosing regimens in certain at-risk breeds [4–16].

In particular, PCR has become a mainstream molecular diagnostic tool in veterinary oncology for several reasons. First, PCR methodologies are relatively inexpensive, which make it a marketable and cost friendly diagnostic test for pet owners–the majority of whom are paying out-of-pocket for veterinary expenses. Second, PCR can be performed with small amounts of easily obtained biologic samples, which is ideal for repeated sampling from the same individual as a function of time and disease status. Third, PCR can be highly sensitive, with the theoretical detection of a single cell when amplification efficiencies are optimized, RNA of high quality, and when the target of interest is disease-specific. While the management of several oncologic processes have advanced following the wide acceptance of clinical molecular testing, continued research in this diagnostic field is necessary to further expand and enhance the screening, monitoring, and treatment of various problematic tumor histologies.

Prostate specific membrane antigen (PSMA) is a membrane protein that serves as a useful molecular diagnostic marker and a potential druggable surface target preferentially expressed by cancer cells or tumor-associated neovasculature [17–19]. PSMA is a transmembrane glycoprotein with multiple enzymatic activities including folate hydrolase and neuropeptidase, which is expressed in both normal and neoplastic prostatic epithelium [20, 21]. Although PSMA expression in extra-prostatic tissues is restricted, it has been shown that intra-tumoral and tumor-associated endothelial cells highly express PSMA [20–22]. Furthermore, in these seminal studies characterizing PSMA expression, non-neoplastic endothelium consistently fail to express PSMA [20–22]. Since its initial description, PSMA overexpression by prostate carcinoma has been leveraged for the development of targeted diagnostic imaging and personalized medicine strategies for patients afflicted with prostate cancer. Diagnostically, PSMA targeting radionuclides have been developed for the sensitive and specific detection of prostatic carcinoma disease recurrence and metastases with PET imaging [23–25]. In parallel, several PSMA-based radioimmunotherapeutics and anti-PSMA antibodies complexed with small molecule inhibitors or chemotherapeutics have completed advanced clinical phase testing and hold promise for improved management of prostatic cancer in men [26, 27].

In addition to its theranostic utility, PSMA has also been explored as a molecular surrogate or quantifiable biomarker for prostatic neoplasia in people using conventional benchtop methods including RT-PCR. Pioneering clinical studies initially demonstrated that RT-PCR for PSMA in whole blood from patients with prostatic pathology was moderately sensitive and highly specific for neoplasia, and this early research showed a correlation between circulating prostatic carcinoma cells and metastasis. Additionally, in a subset of human patients with clinically undetectable prostatic pathology, RT-PCR for PSMA was predictive of future cancer recurrence and metastasis development [28, 29]; findings that highlight the potential value of PSMA amplicon detection for the surveillance of minimal residual disease burden. In complementary studies, other investigative groups have shown that RT-PCR for PSMA and prostate specific antigen (PSA) in peripheral blood was correlated with tumor stage and that elevated amplicon levels were highly predictive of primary tumor extension beyond the capsule of the prostate [30], and similarly PSMA RT-PCR on peripheral blood has been shown to correlated with a higher tumor grade at diagnosis [31]. Lastly, the detection of high levels of PSMA amplicons in blood and bone marrow of patients undergoing radical prostatectomy has consistently shown to be predictive of significantly shorter biochemical disease-free interval than those patients with low PSMA amplicons [32–34]. Collectively, these related clinical investigations solidly support the prognostic value of PSMA amplicon detection in biologic fluids to aid in the diagnosis and management of prostatic carcinoma.

Discrepant with the human oncology field, limited efforts have been focused towards annotating PSMA expressions in naturally-occurring cancers, including prostate carcinoma, arising in companion animals [35–40]. While PSMA appears to be expressed by canine prostate carcinoma, similar to human beings, its expression by other solid tumor histologies and tumor-associated neovasculature remain largely unexplored in veterinary species with the exception of one report describing the expression of PSMA by canine hemangiosarcoma (cHSA) cell lines [41].

Canine hemangiosarcoma is a devastating cancer commonly diagnosed in middle-aged and geriatric patients and represents a malignancy with varying molecular and functional subtypes and principally composed of malignant endothelial cells originating from hematopoietic precursors [42–44]. Clinically, cHSA commonly affects the spleen, liver, heart, skin, and subcutaneous tissues, but can arise from any anatomic location where blood vessels are present [45, 46]. Non-cutaneous and visceral manifestations of this disease are often associated with a poor prognosis due to rapid, disseminated metastasis despite aggressive surgical and medical intervention [46, 47]. Pet dogs with visceral disease commonly present with spontaneous hemoabdomen, which often necessitates exploratory and therapeutic surgical intervention [48, 49]. Pre-operative clinical-based diagnostics that can increase suspicion for cHSA include the presence of hemorrhagic effusion, anemia, and visceral organ mass effects, however, a strongly supportive diagnosis of cHSA is often unachievable with conventional cytopathology [50]. Additional non-invasive tests to increase suspicion for cHSA are reliant upon more specialized techniques or molecular assays including flow cytometry and thymidine kinase 1 detection [42, 51]. While these more advanced diagnostic tests have clear utility in supporting the confirmation of cHSA, the discovery and validation of complementary surrogate biomarkers is warranted to provide veterinary healthcare professionals additional molecular diagnostic tools to bolster confidence in a pre-surgical diagnosis of cHSA or the detection of microscopic disease burden.

Given that PSMA is consistently and differentially overexpressed by neoplastic endothelium in a variety of human solid tumors [20–22], we hypothesize that this surface protein will be overexpressed in cHSA and that the successful characterization of PSMA as malignant endothelial cell marker in cHSA could prove to be a valuable supportive diagnostic biomarker of microscopic disease burden. As such, we sought to characterize gene and protein expression of PSMA in cHSA cell lines as compared to a normal canine aortic endothelial cell line. Furthermore, we aimed to characterize PSMA expression in naturally occurring primary cHSA tumors, associated metastases, and normal tissues. Finally, we developed a discriminatory method by which the detection of PSMA amplicons by qualitative PCR might serve as a surrogate biomarker for the presence of cHSA or other PSMA-expressing cancer cells in dogs with non-traumatic, hemorrhagic body cavity effusions.

## Materials and methods

### Cell lines

Six cHSA cell lines were used in this study including Cindy (provided by Amy MacNeill, Colorado State University), DD1 [52], EmmaBrain [52], EmmaSpleen [52] (provided by Jaime Modiano, University of Minnesota), FITZ [53] (provided by Douglas Thamm, Colorado State University), and SBHSA [54] (provided by Stuart Helfand, Oregon State University). A canine aortic endothelial cell line (CAoEC) was also used to represent non-malignant endothelium (provided by Sue LaRue, Colorado State University). The CPA [55] and ACE-1 [56] cell lines were used as canine positive and negative PSMA controls, respectively (provided by Thomas Rosol, Ohio State University). The LNCaP and PC3 cell lines, derived from human prostate carcinomas, were used as known positive and negative PSMA controls, respectively, and purchased from ATCC (Manassas, VA). All cell lines were cultured in DMEM with 10% fetal bovine serum and 1% penicillin/streptomycin. Cell cultures were maintained in 37°C in 5% CO_2_ and passaged once confluent.

### Reagents and antibodies

A mouse monoclonal, anti-human PSMA antibody (Novus Biological, NBP1-45057, Littleton, CO) was used for western blot analysis and immunohistochemistry (IHC) and a mouse monoclonal, anti-human PSMA antibody (Abcam, ab19071, Cambridge, UK) was used for confocal fluorescent microscopy experiments. An anti-β actin antibody (Abcam) was used as a loading control for western blot analysis. ProLong Gold Antifade Mount (P10144), 4’, 6-diamidino-2-phenylindoleg (DAPI), and goat anti-mouse Alexa 488 secondary antibody (A-11034) were purchased from ThermoFisher Scientific (Waltham, MA). Diva (DV2004), Peroxidazed 1 (PX968), Background Punisher (BP974), secondary antibodies (MC541 and IPK5010), and hematoxylin (CATHE) used in IHC were purchased from commercial vendors.

### RNA collection and PCR techniques

RNA was collected from all cell lines and freshly harvested canine tissues collected at necropsy with a commercially available kit (The Zymo Kit, Cat# R2052, Irvine, CA). Purity of collected RNA, assessed by A260/A280, was performed on a NanoDrop1000 Spectrophotometer (Thermo-fisher, Waltham, MA), and confirmed to be 1.9–2.0 for all samples. One μg of RNA was reverse transcribed to cDNA using Superscript III First-Strand kit (Cat# 18080051) (Thermo-fisher, Waltham, MA).

For qualitative PCR, 5 μL of cDNA was used in combination of 5 μL 10 X PCR buffer, 2 μL of 50 mM MgCl2, 1 μL 10 mM dNTP, 2.5 U of Taq polymerase, 2 μL forward and reverse primers for canine PSMA (5’-gaacgaaacttccagcttgc-3’ and 5’-tcccatatctcgcaatcaca-3’) or β actin (5’- ggcatcctgaccctcaagta-3’ and 5’- ggataccgcatgattccatc-3’) (Integrated DNA Technologies) was added with water to a total reaction volume of 50 μL. The expected amplicon sizes for PSMA and β actin were 361 and 637 base pairs, respectively. Qualitative PCR reactions for both PSMA and β actin were performed on a Peltier thermal cycler (DNA Engine. Bio-Rad) using the following conditions: 95°C 5 minutes, then (95°C 30 seconds, 53°C 30 seconds, 72°C 30 seconds, repeat for cycle 27), then 72°C 7 minutes, then 4°C until analysis.

For quantitative RT-PCR, all PCR reactions were performed in a 96-well reaction plate (Cat# N8010560, Thermo-fisher, Waltham, MA). 5 μL of prepared cDNA was loaded with 1.25 μL canine-specific TaqMan PSMA primer (Cat# 4351372) and 12.5 μL TaqMan fast advanced master mix (Cat# 4444557 Applied Biosystems, Thermo-fisher, Waltham, MA), with a total reaction volume up to 25 μL loaded with the plate being covered with an optical adhesive cover (Cat # 4360954, Thermo-fisher, Waltham, MA). The Applied Biosystems 7500 Real-time PCR thermal cycler was used to run and analyze the PCR reaction (Thermo-fisher, Waltham, MA). For RT-PCR assessment of PSMA in cell lines, 3 independent biologic replicates were performed, with 5 technical replicates within each independent experiment being performed for each cell line.

### Cell protein collection

Cells were grown to 80–90% confluence, rinsed with PBS, then trypsinized and centrifuged at 450 g for 5 minutes at 4°C. Cell pellets were resuspended in 1 ml PBS, centrifuged at 1,100 g for 5 minutes at 4°C, and supernatant removed. The resultant cell pellet was resuspended with 100 μL of Mammalian Protein Extraction Reagent, mixed with protease inhibitor cocktail solution for 15 minutes, and then centrifuged at 1,100 g for 10 minutes at 4°C. Resultant protein lysates were assessed for respective concentrations using a standard assay kit (BCA Protein Assay kit, Pierce).

### Western blot analysis

Protein lysates (5–50 μg) collected from cell lines were electrophoresed on a 12% polyacrylamide gel, transferred to a nitrocellulose membrane, and block with tris buffered saline-tween 20 (TBST) with 5% nonfat dry milk (NFDM) for 1 hour at room temperature. Western blot analysis was performed using a mouse monoclonal, anti-human PSMA antibody (Novus Biological, NBP1-45057, Littleton, CO) at a concentration of 1:250 in TBST with 5% NFDM, incubated for 1 hour at room temperature. The membrane was then washed 3 times with TBST, probed with a rabbit anti-mouse secondary antibody diluted 1:5000 in TBST with 5% NFDM, and developed using ChemiDoc XRS+ molecular imager system. Band intensity analysis was done using Image Lab software. Relative protein expressions were adjusted against β-actin using anti-human antibody at a concentration of 1:5000 in TBST with 5% NFDM, incubated for 1 hour at room temperature. Results reported were derived from at least 2 independent experiments.

### Confocal fluorescent microscopy

Cells were seeded at 10^4^ cells per well in chamber well slides and incubated overnight. Following incubation, cells were washed with phenol red-free DMEM, and fixed with 4% methanol-free paraformaldehyde for 20 minutes at room temperature. Cells were permeabilized for 10 minutes at room temperature using 0.1% Triton X-100. Cells were then washed with PBS, blocked with 3% bovine serum albumin (BSA) in PBS for 45 minutes at room temperature and then rinsed with PBS. A mouse monoclonal anti-human PSMA antibody (Abcam, ab19071, Cambridge, UK) at a concentration of 1:500 (in 3% BSA in PBS) was incubated with fixed cells for 24 hours at 4°C, then counterstained with DAPI for 10 minutes. Cells were incubated with Alexa Fluor 488 goat anti-mouse antibody at a concentration of 1:100 (in 3% BSA in PBS) for 60 minutes at room temperature while protected from light. Cells were washed with PBS and mounted with 5 drops of ProLong Gold solution, and left to dry at room temperature for 24 hours protected from light. Cells were imaged using a Ziess LSM 700 confocal laser scanning microscope, and image analysis performed with ImageJ software. PSMA stain intensity was derived from 25–50 individual cell counts/well and expressed as fluorescence intensity per surface area (RFU/μm^2^). A total of 2 independent experiments were conducted.

### Immunohistochemistry

Thirty-four formalin-fixed, paraffin-embedded (FFPE) primary and metastatic spontaneous cHSA tissue blocks were retrieved from the University of Illinois’ Veterinary Diagnostic Laboratory for immunohistochemical assessment. Following tissue sectioning, slides underwent a standardized deparaffination procedure (xylene, 3 minutes x 3; 100% ethanol, 1 minute x 2; 95% ethanol, 1 minute x 1; 70% ethanol, 1 minute x 1; running water, 1 minute x 1). Slides were treated with Diva Decloaker and then blocked with Peroxidazed 1 for 5 minutes. A secondary block with Background Punisher was performed for 10 minutes. Slides were then incubated with a mouse monoclonal anti-human PSMA antibody (Novus Biological, NBP1-45057, Littleton, CO) at a concentration of 1:1000 for 30 minutes. Slides were incubated with a biotinylated secondary antibody for 30 minutes then with the chromogen IP FLX DAB. Slides were counterstained with hematoxylin (Cat hematoxylin). All stains and reagents used for immunohistochemistry were obtained from Biocare Medical (Pacheco, CA). All samples were evaluated by a single anatomic pathologist (JPS) and relative PSMA staining intensity was numerically scored using a previously published protocol [57]. This protocol assigns a score of “0” for no staining; a score of “1” for weak staining in ≤ 50% of cells; a score of “2” for strong staining in ≤ 50% of cells or weak staining in > 50% of cells; and a score of “3” for strong staining in > 50% of cells. In addition to naturally occurring tumor samples, PSMA staining was evaluated in a normal canine tissue microarray with 28 distinct tissue samples (DGF281, US Biomax, Inc.).

### Optimal PCR protocol and dynamic range for qualitative PSMA amplicon visible detection

In order to determine the optimal PCR cycle number capable of preferentially detecting PSMA in cHSA but not in normal endothelium, the FITZ (lowest PSMA-expressing cHSA cell line) and CAoEC cell lines were compared head-to-head across a range of thermocycles ranging from 24–30 for visible production of PSMA amplicons. Quantitative comparisons across different thermocycles were expressed as ratios [FITZ (PSMA/β actin)]/[CAoEC (PSMA/β actin)], with increasing ratios corresponding to superior differential amplification of PSMA between FITZ and CAoEC cell lines.

After the optimal number of amplification cycles was determined, the dynamic range of qualitative PCR for generating visible target amplicons from the lowly-expressing PSMA FITZ cell line was evaluated. The FITZ cell line, ranging from 10^0^ to 10^7^ cells, was tested for the ability to produce visible PSMA amplicons when spiked into PBS (pure FITZ) or into 2 ml of EDTA whole blood (FITZ + nucleated cells). In experiments where FITZ cells were spiked into whole blood, the cell mixture was vortexed and diluted 1:10 with red blood cell lysis buffer (Sigma-Aldrich) for 5 minutes. Subsequently, cell mixtures were centrifuged at 800 g for 5 minutes and the red blood cell lysates were discarded. The resultant cell pellets were then mixed with 1 ml of RNALater solution (ThermoFisher) and stored at -80°C until analysis. Qualitative PCR at 27 cycles for PSMA and β actin were performed, and ratios expressed as PSMA/β actin (pixel/area). A total of 3 independent experiments were performed. An identical sample handling and red blood cell lysis protocol described above was used to explore the detection of PSMA amplicons in EDTA whole blood collected from 10 healthy dogs.

### Tandem molecular and cytologic detection of microscopic disease

Hemorrhagic effusions from the abdominal cavity of three dogs and from the pericardial sac of two dogs, all with stage II or III disease (macroscopic primary tumor > 5 cm or ruptured), were collected and stored at 4°C until fluid analysis was performed within 6 hours of collection. A buffy coat slide was prepared from the effusions to concentrate the nucleated cells. A 500 cell differential was performed and included neutrophils, lymphocytes, macrophages, eosinophils, basophils and atypical cells. Atypical cells included those cells exhibiting criteria of malignancy which could represent the neoplastic population or highly reactive mesothelial cells. Slides were stained with Wright-Giemsa stain and reviewed by a single clinical pathologist (AMB).

Concurrently with cytopathological analysis, 2 mL of hemorrhagic effusion were collected in EDTA tubes from these 5 patients at the time of presentation. Using the previously described methods, red blood cells were lysed, RNA was collected, cDNA was synthesized, and qualitative PCR was performed at the optimized cycle number (27 cycles) to assess for the detection of PSMA amplicons presumable derived from rare cHSA cells. All studies performed with pet dogs were approved by the Institutional Animal Care and Use Committee (#18133).

### Statistical analysis

For PSMA gene and protein expressions in cell lines evaluated, 1-way ANOVA was used to evaluate for differences in basal PSMA expressions with the use of Dunnet’s post-hoc test. Statistical calculations were performed using GraphPad Instat3, and *p* < 0.05 was considered statistically significant for all analyses.

## Results

### Quantitative differences in basal PSMA gene expression in cell lines

**Fig 1:**
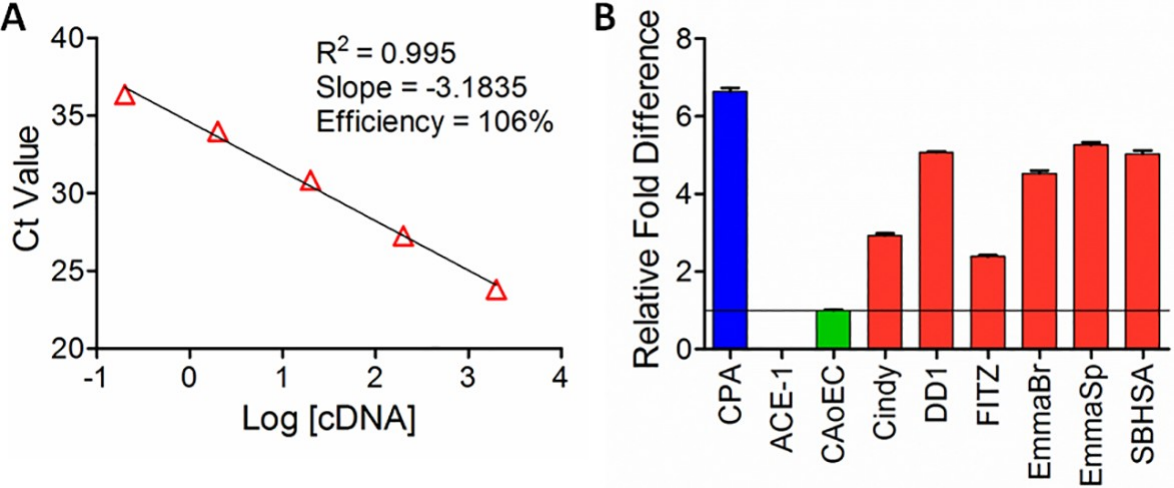
Gene transcription of PSMA across cell lines. (A) Validation of canine-specific TaqMan PSMA primer efficiency across 5-log orders of RNA concentration using CPA, a canine prostatic carcinoma cell line. (B) Comparative PSMA gene expression across canine cell lines. Relative PSMA expressions for six cHSA cell lines (red) compared to the non-malignant canine endothelial cell line, CAoEC (green). Positive and negative control cell lines include CPA (blue) and ACE-1 (purple), respectively. Representative data presented from 3 independent biologic replicates with 5 technical replicates for each cell line.

The efficiency of TaqMan primers for canine PSMA was determined by using CPA as a positive canine control cell line. Five log orders of CPA RNA concentrations (0.032–320 ng) were run in quintuplicate and Ct values determined by RT-PCR. Ct values were plotted against log cDNA concentration and fitted by linear regression for the calculation of slope and linearity (R^2^) (Fig 1A). RT-PCR primer efficiency was calculated to be 106% using the formula:

$$Efficiency=‑1+10^{\left(‑1/slope\right)}.$$

After validating primer efficiency, relative PSMA gene expression in control and cHSA cell lines were normalized against CAoEC (Fig 1B). Highest relative PSMA gene expression was noted in the positive control cell line (CPA, 6.64 ± 0.21), while the negative control (ACE-1) had nearly undetectable PSMA gene expression (0.01 ± 0.00). Relative PSMA expressions among cHSA cell lines were variable ranging from 2.4–5.3 times greater than CAoEC expression. The average relative PSMA gene expressions in cHSA cell lines compared to the CAoEC cell line were as follows: Cindy 2.93 ± 0.15, DD1 5.08 ± 0.04, FITZ = 2.39 ± 0.08, EmmaBrain 4.53 ± 0.17, EmmaSpleen 5.26 ± 0.15, and SBHSA 5.03 ± 0.19.

### Validation of antibody cross-reactivity for canine PSMA

**Fig 2:**
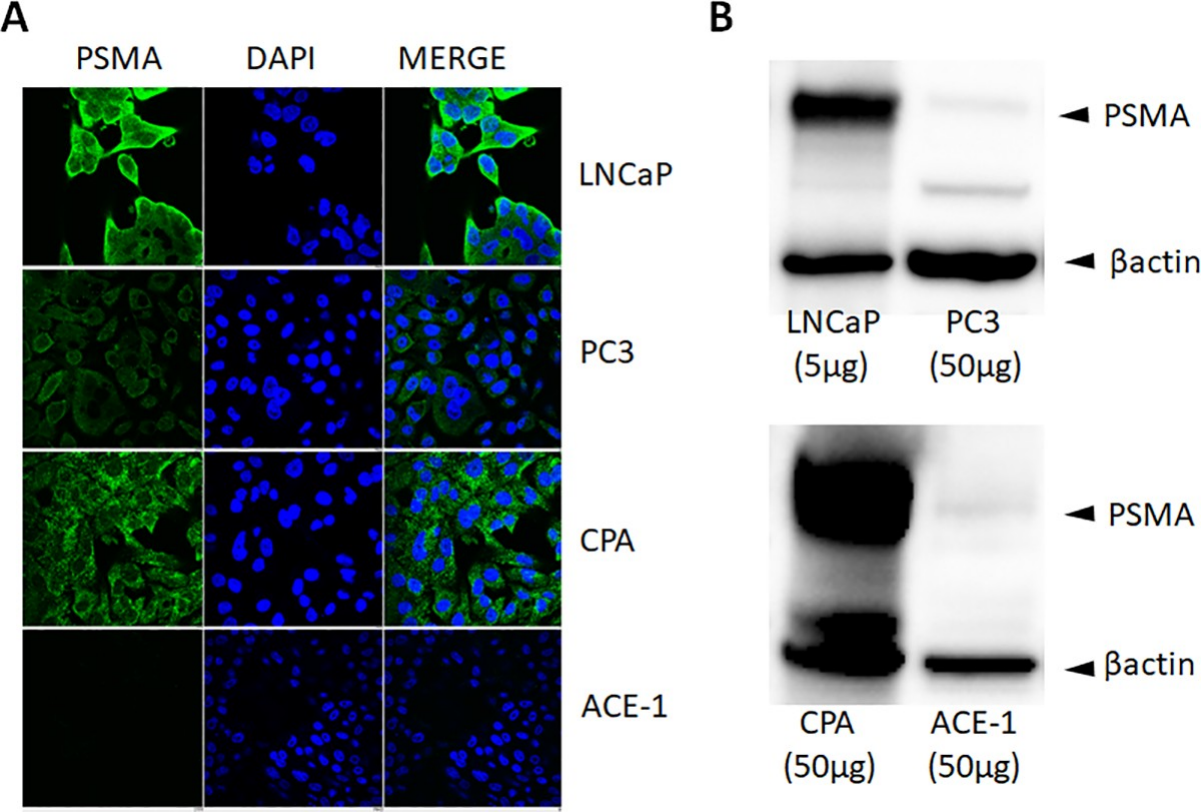
Antibody validation for canine PSMA protein expression. Validation of antibodies for detecting PSMA in human and canine cell lines by (A) confocal fluorescent microscopy, and (B) western blot analysis. Positive and negative controls for canine and human include (CPA and ACE-1) and (LNCaP and PC3), respectively.

Two mouse monoclonal anti-human PSMA antibodies, (Abcam, ab19071, Cambridge, UK and Novus Biological, NBP1-45057, Littleton, CO) were validated for canine cross-reactivity by confocal fluorescent microscopy (Fig 2A) and western blot analysis (Fig 2B), respectively. Predicted and appropriate protein expressions were noted in both human and canine cell lines, inclusive of both positive and negative controls.

### Comparison of PSMA expression across normal and malignant endothelial cell lines

**Fig 3:**
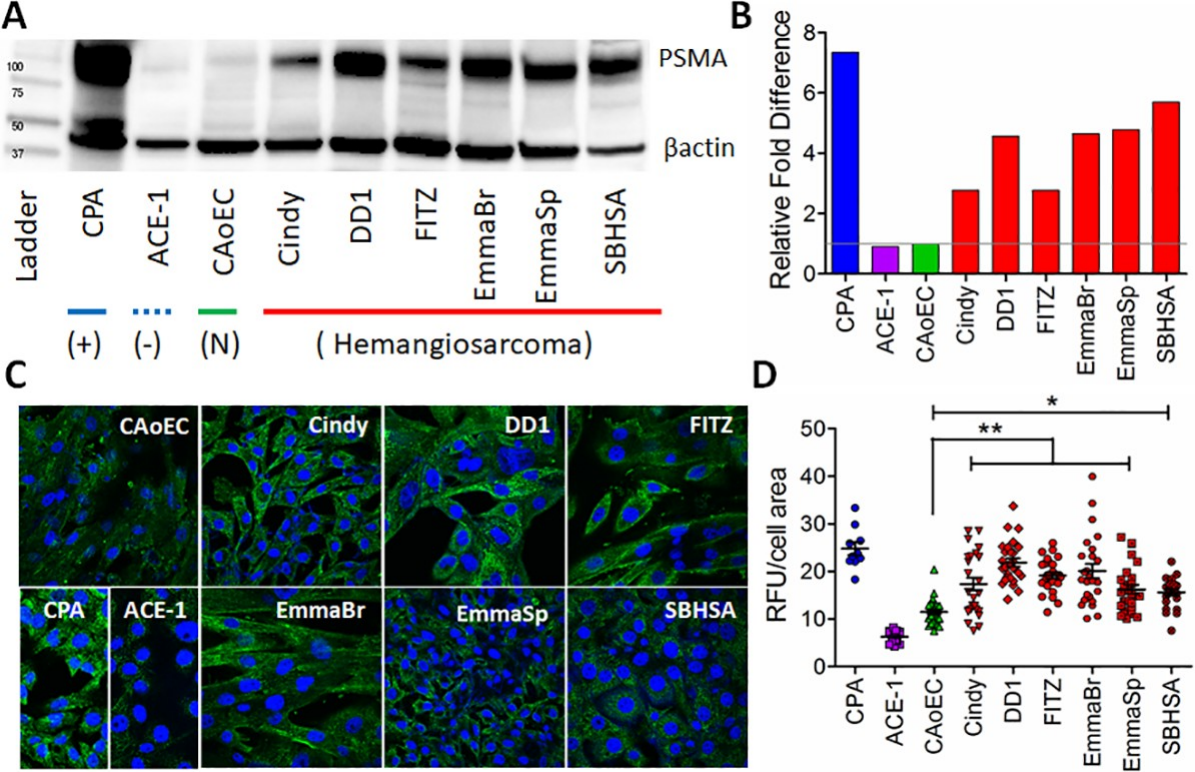
Comparative PSMA protein expressions across cell lines. Comparison of PSMA protein expressions and associated histograms for six cHSA cell lines (red) relative to the non-malignant CAoEC line (green) by (A and B) western blot analysis, and (C and D) by confocal fluorescent microscopy. Canine positive and negative controls are CPA (blue) and ACE-1 (purple), respectively. Significance *p* < 0.05 and *p* < 0.01 denoted by “*” and “**”, respectively.

PSMA protein expression was assessed across normal and malignant endothelial cell lines. By western blot analysis, all six immortalized cHSA cell lines overexpressed PSMA relative to the non-malignant endothelial cell line, CAoEC (Fig 3A and 3B). Though PSMA expressions across cHSA cell lines were variable with the FITZ and CINDY cell lines being the lowest expressers (2.8 relative fold increase) and SBHSA being the highest (5.7 relative fold increase), the collective panel of cHSA cell lines demonstrated an average increase in PSMA by 4.2 fold compared with CAoEC. Similarly, confocal fluorescent microscopy also consistently trended with increased PSMA expressions in cHSA cell lines relative to CAoEC (Fig 3C and 3D), although the magnitude of differences were less than identified by western blot analysis. By confocal fluorescent microscopy, the fold elevations for PSMA expressions in cHSA cell lines relative to CAoEC ranged from 1.4 to 1.9, and reached statistical significance for all comparisons.

### Expression of PSMA in spontaneous cHSA tumors

**Fig 4:**
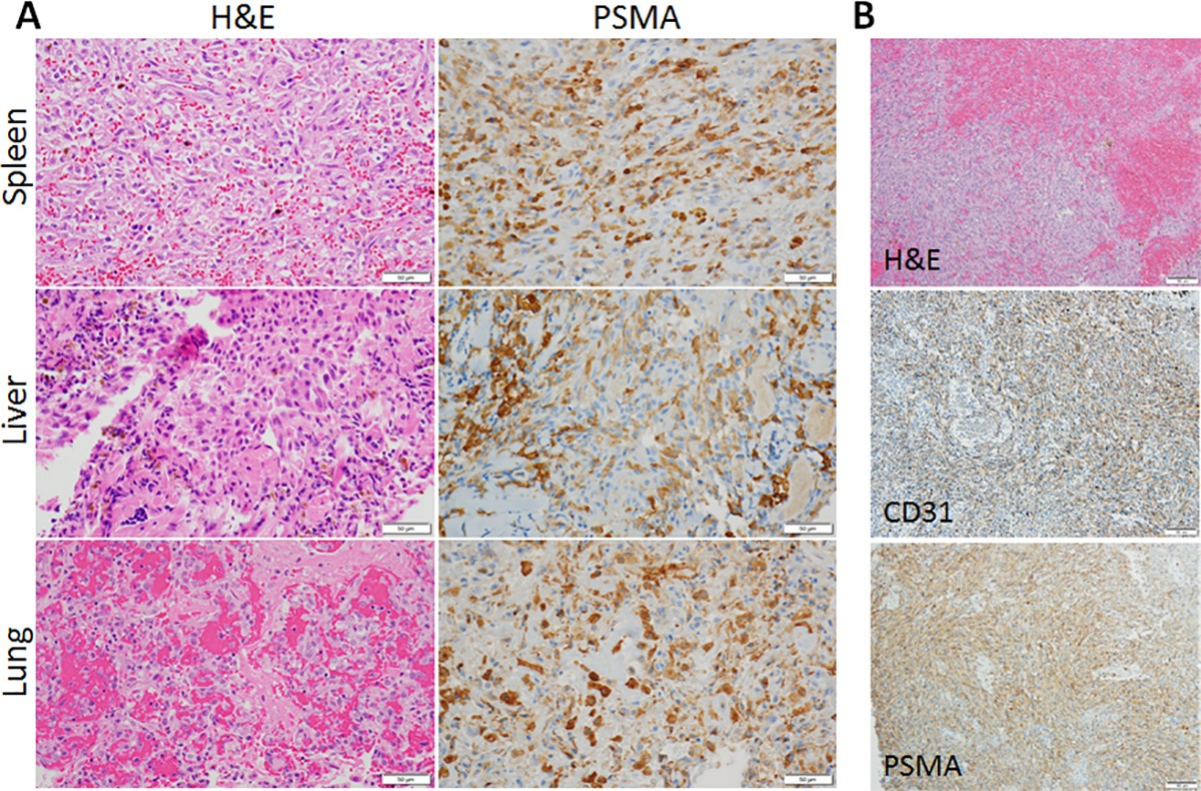
Characterization of PSMA in cHSA tumor samples. Spontaneously-arising cHSA tumor samples involving the spleen, liver, and lung tissues, with representative (A) histology by H&E and PSMA immunohistochemistry (Score 3; magnification 400x), and comparison (B) of staining properties for cHSA by CD31 and PSMA (magnification 100x).

PSMA immunohistochemistry (IHC) was performed on spontaneous primary and metastatic cHSA samples from 27 distinct canine patients (total of 34 tissue samples). PSMA staining intensity was variable and heterogeneous across and within tumor samples; however, positive PSMA expression was noted in all cHSA samples (Fig 4A, S1 Fig). Assignment of PSMA IHC scores for cHSA samples were the following: Spleen- score 1 (n = 4), score 2 (n = 9), and score 3 (n = 2); Liver- score 1 (n = 5), score 2 (n = 5), and score 3 (n = 1); and Lung- score 1 (n = 2), score 2 (n = 5), and score 3 (n = 1). Immunohistochemical staining characteristics for PSMA in spontaneously-arising cHSA tissue samples were analogous to a more conventional endothelial marker, CD31 (Fig 4B).

### Characterization of basal PSMA expressions by IHC and RT-PCR in normal canine tissues

PSMA gene and protein expression were evaluated in normal canine organs to characterize basal expression patterns across diverse tissue types. Using a commercial canine tissue microarray, 28 distinct cores were stained for PSMA by IHC. Corroborating data published in the Human Protein Atlas project, PSMA was notably expressed by various organs, in particular kidney, liver, and glandular epithelium lining the intestinal tract (S2 Fig). Rarely were IHC PSMA staining patterns in the canine tissue microarray discrepant from what has been reported in humans, with the exception of robust PSMA immunoreactivity in thin walled capillary vessels making up the pulmonary alveolar spaces in canine lung specimens, a positive staining pattern absent in the Human Protein Atlas project. To corroborate the observed PSMA protein expressions in normal tissues, RT-PCR using canine-specific TaqMan PSMA primers was performed on freshly collected canine tissues (S3 Fig), with PSMA gene transcription results approximating the observed trends identified by IHC methodologies.

### Optimal PCR methodology and dynamic range of PSMA gene expression

**Fig 5:**
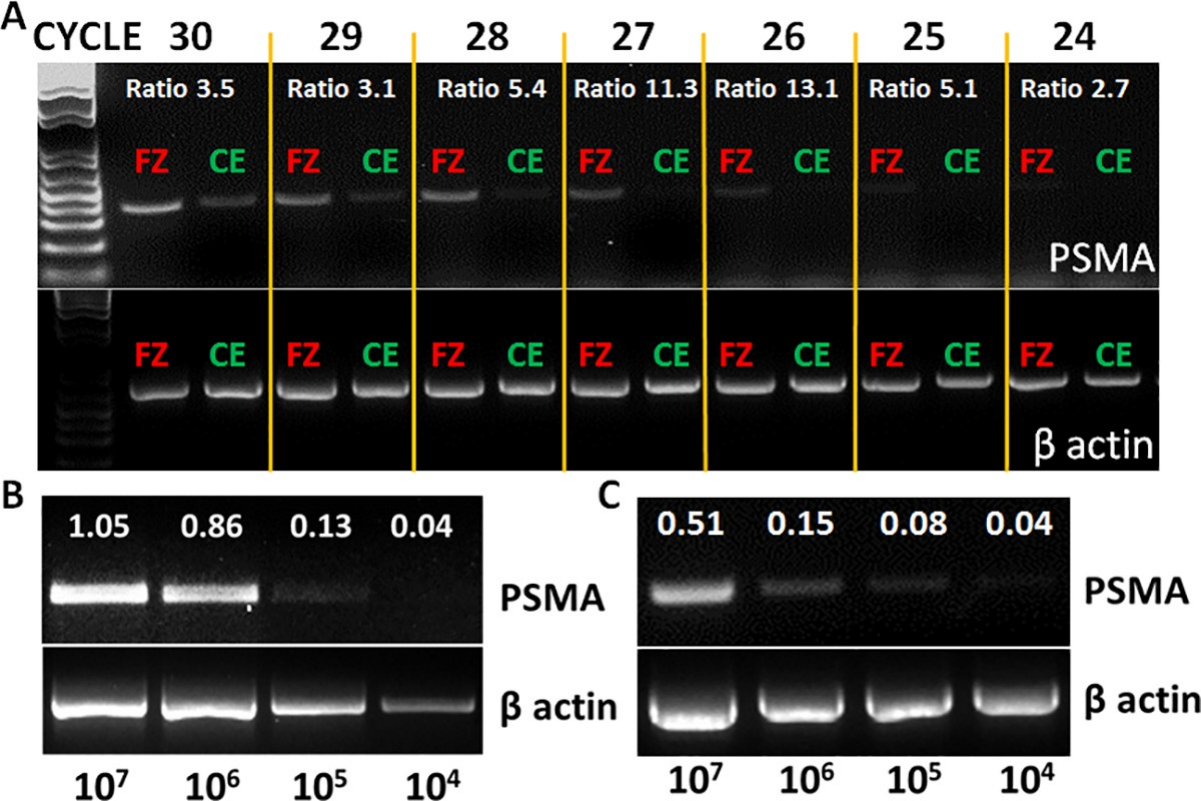
Optimization of qualitative PCR methodology and dynamic range of detection for PSMA. (A) Differential PSMA amplicon generation by qualitative PCR with the lowly expressing PSMA cHSA cell line (FITZ; red) compared with CAoEC (green) across different thermocycles ranging from 24 to 30. Determination of qualitative PCR sensitivity for detecting PSMA amplicons at 27 cycles with a logarithmic titration of (B) FITZ cells only or **(C)** when FITZ cells are spiked into whole blood. Lowest cell density capable that produces faintly visible amplicons is 10^4^ FITZ cells, alone or in addition to whole blood nucleated cells. Amplicon production expressed as a ratio of PSMA/β actin pixels/area. Abbreviations FITZ (FZ; red) and CAoEC (CE; green).

The preferential detection of PSMA amplicons by qualitative PCR in FITZ (lowest PSMA expressing cHSA cell line), as compared to CAoEC, was optimally achieved between 26–28 cycles, with normalized ratios of FITZ/CAoEC being 13.1 at 26 cycles, 11.3 at 27 cycles, and 5.4 at 28 cycles (Fig 5A). At 27 cycles, the dynamic range for visually detecting PSMA amplicons produced by pure FITZ cells or FITZ cells spiked into 2 mL of EDTA whole blood was observed to be minimally between 10^4^ to 10^5^ cells (Fig 5B or 5C, respectively). For FITZ cells spiked into EDTA whole blood, the minimal number of 10^4^ FITZ cells required to produce weakly visible PSMA amplicons calculated to a detection limit of 1 cHSA cell per 2,000 nucleated cells (assumption of 10^4^ white blood cells/μL for whole blood). In ten healthy normal dogs, 2 mL of EDTA whole blood and associated nucleated cells fail to produce any visible PSMA amplicons after 27 cycles of amplification, contrasting with the positive control cell line (CPA), which clearly produces visible PSMA amplicons (S4 Fig).

### Detection of PSMA amplicons in hemorrhagic effusions collected from dogs with cHSA

**Fig 6:**
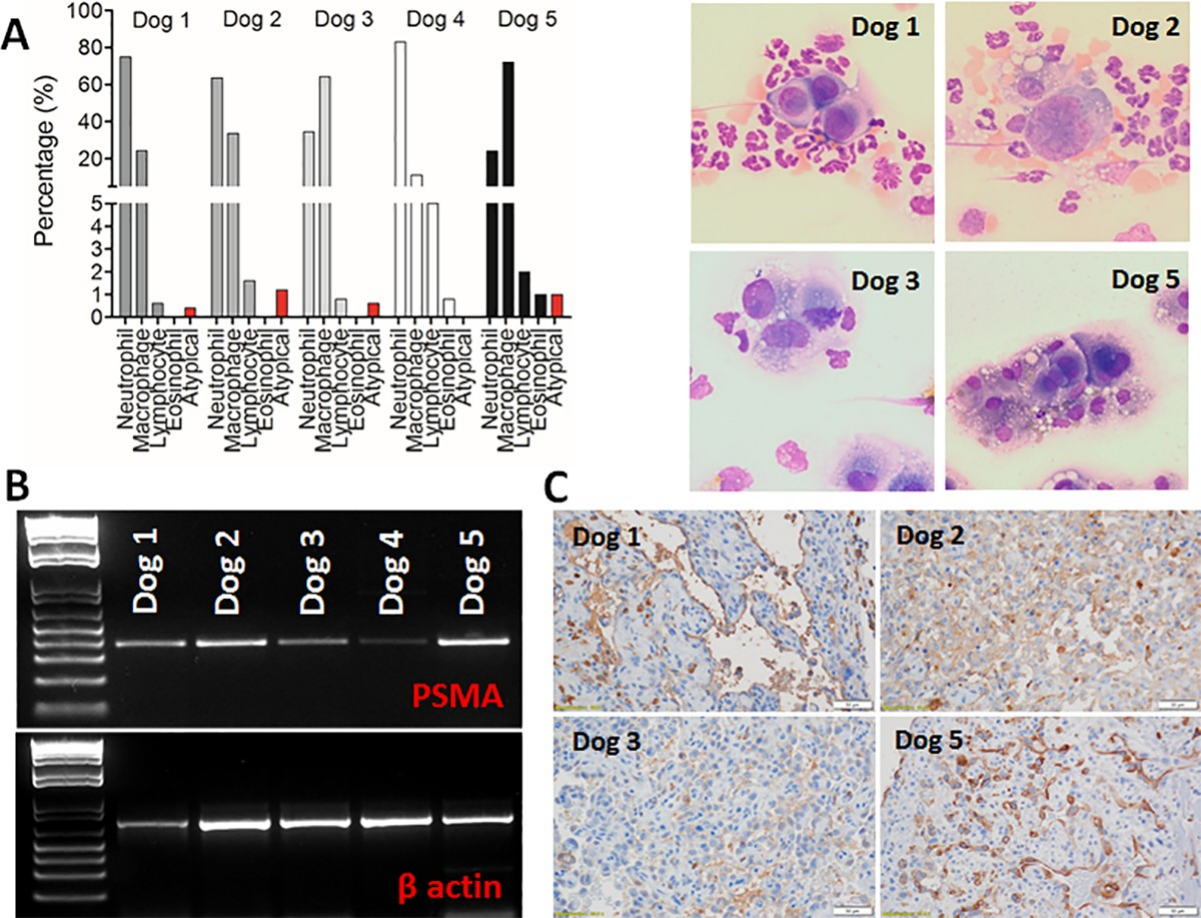
Tandem cytologic evaluation and PSMA amplicon generation from hemorrhagic effusions. (A) Cytologic characterization of hemorrhagic effusions collected from five dogs, three with hemoabdomen (Dogs 1, 2, and 4) and two with hemorrhagic pericardial effusion (Dogs 3 and 5). Note the identification of atypical cells in Dogs 1–3 and 5 (right, top panel). (B) Generation of visible PSMA amplicons (27 cycles) from hemorrhagic effusions overtly positive for Dogs 1–3 and 5, and weakly positive for Dog 4. (C) Immunohistochemical evaluation of primary tumors from Dogs 1–3 and 5, confirming that primary tumors presumed to be the source of exfoliative cHSA cells stain positively for PSMA.

**Fig 7:**
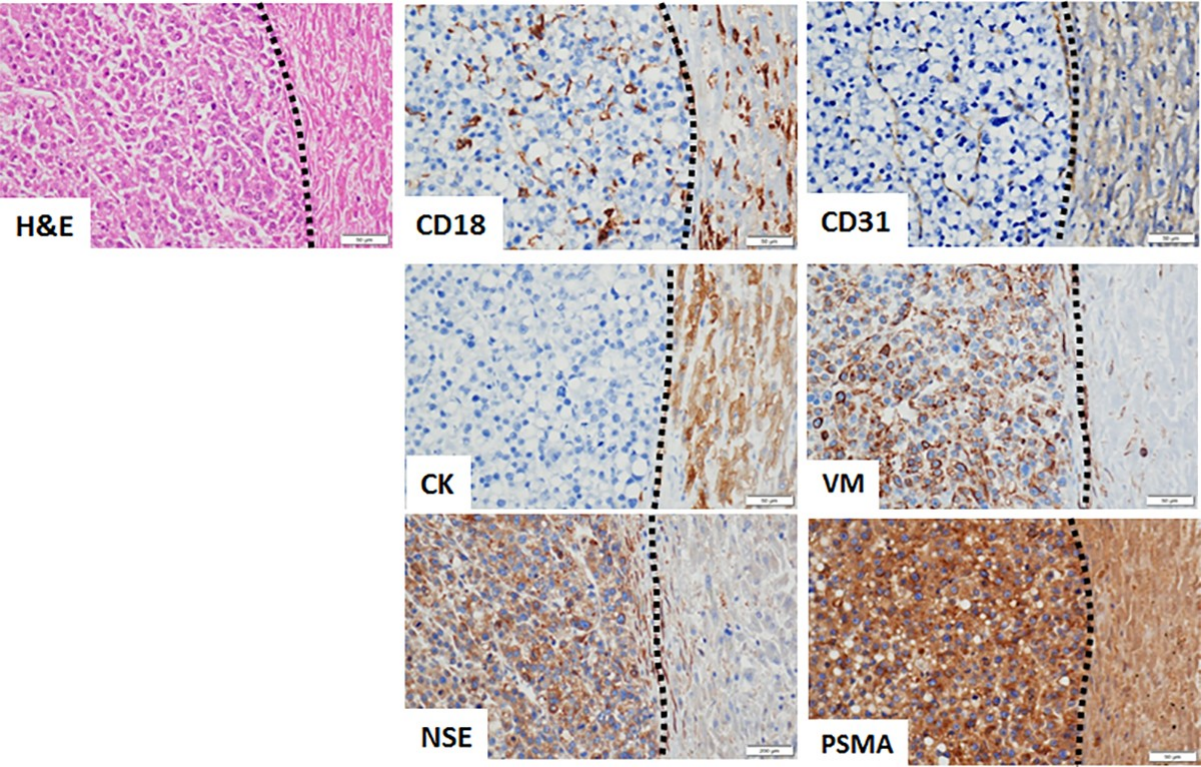
Generation of PSMA amplicons from non-cHSA tumor histology. Immunohistochemical evaluation of tumor from Dog 4, showing immunopositivity for vimentin, neuron-specific enolase, and PSMA only. Dotted black line separates normal liver tissue (right) from tumor tissue (left). CD18 and CD31 immunohistochemistry fail to demonstrate immunoreactivity within tumor cells, but do stain resident macrophages and normal blood vessels, respectively. Magnification 400x; abbreviations CK, cytokeratin; VM, vimentin; NSE, neuron-specific enolase.

Conventional cytologic evaluation of hemorrhagic effusions collected from pet dogs identified a low percentage (0.4–1.2%) of atypical cells in most patients (Dogs 1–3 and Dog 5; Fig 6A). Atypical cells were distinct from reactive mesothelial cells and possessed cytologic criteria of malignancy (anisocytosis, anisokaryosis), however extremely low numbers of cells identified within samples precluded definitive cytologic diagnoses. Correspondingly, PSMA amplicons derived from hemorrhagic effusions could be robustly detected by qualitative PCR for Dogs 1–3 and Dog 5, and weakly for Dog 4 (Fig 6B). For Dogs 1–3 and Dog 5, corresponding with PSMA amplicon detection, the primary tumors responsible for shedding tumor cells within hemorrhagic effusions were confirmed to be cHSA (S5 Fig) and PSMA positive (Fig 6C). Histologic classification of Dog 4’s tumor was not consistent for cHSA (CD31 immunonegativity; Fig 7), but the identification of faint PSMA amplicons for Dog 4, coincide with tumoral immunohistochemical reactivity for vimentin, neuron-specific enolase, and PSMA (Fig 7), suggestive of an atypical hepatic carcinoid tumor.

## Discussion

The validation of surrogate biomarkers that have the capacity for differentiating between normal and pathologic disease states has potential to provide theranostic utility for various maladies including cancer. In the current report, the expressions of PSMA were investigated across a panel of endothelial-derived cell lines and tissue samples, both normal and malignant, to assess if PSMA expressions could be leveraged for aiding in the diagnosis of cHSA, a highly metastatic solid tumor affecting companion animals [45, 47]. Malignant cHSA cells expressed PSMA up to 6x greater at both gene and protein levels compared to a normal endothelial cell line, and this observed differential expression provided the opportunity to develop a qualitative PCR methodology that allowed for the preferential detection of PSMA amplicons within hemorrhagic effusions containing low percentages (≤ 1%) of rare atypical cells, presumably of cHSA origin. Importantly, the detection of PSMA amplicons were not detectable in EDTA whole blood samples collected from normal healthy dogs, suggesting that the qualitative PCR methods developed and used for PSMA amplification have the capacity to discriminate between the presence or absence of cHSA cells within biologic fluids.

Molecular diagnostics that detect PSMA amplicons within blood or tumoral PSMA expression are considered integral components for the diagnosis, prognostication, and monitoring of disease progression associated with high grade prostatic carcinoma in humans [28–34, 58–63]. Analogous to these foundational prostatic carcinoma studies conducted in men where PSMA expressions contribute to patient management, the preliminary findings reported in the current investigation are highly provocative, and suggest that PSMA amplicon detection in hemorrhagic effusions derived for pet dogs might also provide clinically useful information for assisting in the non-invasive and supportive diagnosis of cHSA or other PSMA-expressing cancer cells passively shed into the local microenvironment. Intriguingly, in the five dogs evaluated for hemorrhagic effusion (3 peritoneal and 2 pericardial), a histologic diagnosis of cHSA was confirmed post-mortem in four patients (Dogs 1–3 and Dog 5), and were in agreement with the atypical cells identified on cytology and positive generation of PSMA amplicons derived from hemorrhagic effusions. Correlatively, the primary tumors, which presumable serve as the source of exfoliative cHSA cells and associated PSMA amplicons, were likewise confirmed to express PSMA. Discrepant from the others, Dog 4 that presented for hemoabdomen secondary to a bleeding liver mass, histologically did not have a diagnosis consistent with cHSA (CD31 negative), and likewise did not have any atypical cells identified on cytology. Interestingly, a weak PSMA amplicon signal was detectable, and coincided with the liver mass staining positively for vimentin, neuron-specific enolase, and PSMA. Based upon the panel of immunohistochemical markers, Dog 4 likely had an atypical hepatic carcinoid tumor. Taken singularly, this case example would suggest that PSMA amplicon detection could be more sensitive than cytology and have broader applications for supporting the diagnosis of other tumor types additional to cHSA. Derived from this small cohort of dogs evaluated, PSMA amplicon detection from hemorrhagic effusions might provide supplemental information for aiding in the diagnosis of cHSA or other PSMA-expressing cancers when malignant cells are exfoliative in nature.

While PSMA expressions were definitively divergent between immortalized endothelial cell lines of malignant and non-malignant origin, the immunohistochemical detection of PSMA in naturally-occurring cHSA samples and normal tissues were variable and overlapping. Of the 34 cHSA tissue samples evaluated, all samples demonstrated some degree of PSMA immunoreactivity, with 11/15 splenic, 6/11 hepatic, and 6/8 lung cHSA samples staining moderately (score 2) to strongly (score 3) positive. However, as predicted by the Human Protein Atlas project, gene transcription and immunohistochemical staining for PSMA was robustly identified in several solid visceral organs (kidney, liver, prostate), as well as intestinal glandular epithelium. One notable difference between human and canine tissues was the high level of PSMA RNA and protein identified in canine lung tissue, but nearly completely absent in human beings. Given the expression of PSMA in a limited number of normal canine tissues, it should be argued that results for positive PSMA amplicon detection in hemorrhagic effusions could be confounded by the origin of bleeding. As such, additional studies should be conducted to determine if the described PCR methodology developed and piloted in this investigation can reliably discriminate, with high sensitivity and specificity, among different categories of abdominal hemorrhage and hemorrhagic effusions of non-neoplastic and neoplastic processes.

While not specifically investigated in the current study, the utility of detecting cHSA or PSMA-positive malignant cancer cells in systemic circulation could provide a non-invasive method to identify micrometastatic disease, as well as to serially monitor for disseminated disease progression; analogous to the quantification of PSMA amplicons in circulating blood in men diagnosed with prostatic carcinoma [28, 29]. Although blood collected from normal healthy dogs failed to sufficiently amplify visible PSMA amplicons, the clinical significance for PSMA detection from whole blood will require thorough prospective studies across different disease settings. In the context of cHSA, translational advancement of the described PCR methodology will require evaluating the sensitivity and specificity of PSMA amplicon detection as an accurate surrogate of circulating cHSA cells in the peripheral blood of dogs with disseminated disease. However, interpretative caution should be exercised when PSMA amplicons are detected in suspect patients given the possibility for confounding variables. Of particular importance is the potential presence of circulating endothelial cells (CECs), which are thought to be variably present in the blood of humans afflicted with a wide arrange of diseases including but not limited to sepsis, vasculitis, cardiac and renal disease [64, 65]. In dogs, although no specific studies have evaluated CECs across diverse pathologic conditions, a recent study detected CECs in the blood of healthy dogs, with an average of ~ 40 CECs/mL of whole blood [66].

While the focus of the study was to characterize PSMA as an ancillary diagnostic marker for cHSA, the confirmed overexpression of PSMA in cHSA also raises the potential for it to serve as a druggable target. Such is the case in human oncology, whereby clinical research is ongoing with PSMA-targeted therapies, which include anti-PSMA monoclonal antibodies, monoclonal antibodies conjugated to cytotoxins or radionuclides, and small molecule inhibitors [17, 67–70]. While veterinary translational studies have not yet evaluated novel anticancer strategies that target PSMA positive cancers, preclinical studies utilizing a cHSA xenograft murine model and a PSMA-targeting strategy have been reported [41]. In this preclinical investigation, the administration of doxorubicin-containing nanoparticles decorated with PSMA-targeting aptamers resulted in the preferential delivery of therapeutic nanoparticles into the tumor microenvironment with consequent macroscopic tumor regression. Importantly, despite the expression of PSMA by normal tissues, no histologic evidence of organ toxicity was noted.

While the results of this investigation are intriguing and offer promise for supporting the diagnosis of cHSA through the detection of PSMA amplicons in hemorrhagic effusions, the PCR methodology developed and utilized in this study likely has a narrow operational range given the non-dichotomous expressions of PSMA by normal tissues. To discern the presence of cHSA cells by qualitative PCR, yet maintain background, non-malignant PSMA expressing cells below the level of detection, the optimal number of cycles was determined to be 26–28. This low cycle number limits the production of highly visible amplicons, and thereby diminishes visual acuity for classifying an unknown sample as being positive or negative. Furthermore, because PSMA is relatively weakly expressed even by cHSA cells, the ability to identify PSMA amplicons at 26–28 cycles was limited (minimally 10^4^ cHSA cells spiked into 2 mL of whole blood), requiring a cell density of 1 cHSA for every 2,000 nucleated cells for minimal visible amplicon detection. As such, hemorrhagic effusions with lower malignant cell concentrations would likely be identified as falsely negative, despite the presence of microscopic, minimal residual disease burdens. Finally, the added value of detecting PSMA amplicons from hemorrhagic effusions for supporting the diagnosis of cHSA would be less impactful in samples where definitive cytologic evidence for malignancy has already been identified.

## Conclusions

The data generated in this study demonstrates that differential gene and protein expressions of PSMA exists between normal and malignant endothelial cell populations and affords the feasibility to discriminate between these 2 cellular phenotypes by the measurement of PSMA using conventional molecular techniques. Furthermore, PSMA can be considered an additional immunohistochemical marker to support the diagnosis of cHSA on formalin-fixed tissue samples. Finally and most provocatively, detection of PSMA amplicons from hemorrhagic effusions might provide ancillary, yet valuable, supportive information to aid in the non-invasive and supportive diagnosis of cHSA. However, the definitive diagnosis of cHSA should be derived from the aggregate of clinical, pathological, and molecular determinants, and reliance upon a singular disease surrogate, such as the detection of PSMA amplicons, would be imprudent given its non-selective expressions across multiple tissue histologies. Although further studies are required to better define the sensitivity and specificity of this qualitative PCR methodology, as well as to evaluate the utility of complementary methods such as quantitative RT-PCR, RNA-Seq, and whole exome sequencing, the foundation has been constructed through the conductance of this initial report and provides the platform to assess the clinical significance and utility of PSMA amplicons in dogs with cHSA and other PSMA-expressing cancers.

## Supporting information

S1 Fig PSMA immunohistochemistry from spleen, liver, and lung cHSA samples. Representative H&E and PSMA immunoreactivity (scores 1 and 2) of selected cHSA tissue samples involving spleen, liver, and lung parenchyma.
(TIF)

S2 Fig Normal tissue microarray staining for PSMA. Representative H&E and PSMA immunoreactivity of select canine tissues derived from a commercial normal canine tissue microarray containing 28 cores. Confirmation of expected PSMA immunoreactivity based upon Human Protein Atlas data, with the exception of lung, which is strongly positive in canine only.
(TIF)

S3 Fig Normal tissue gene transcription for PSMA. Comparison of Ct values generated by RT-PCR and canine-specific TaqMan PSMA primer for CPA (positive control; blue), FITZ (low PSMA expressing cHSA; red), and normal canine tissues.
(TIF)

S4 Fig Characterization of PSMA expression in blood of normal healthy dogs. Evaluation of PSMA amplicon generation by qualitative PCR methodology (27 cycles) using 2 mL of EDTA whole blood collected from 10 healthy, young pet dogs (K9#1–10). No visible amplicons produced from whole blood of healthy dogs. CPA serves as positive PSMA control in lanes 1, 7, and 13.
(TIF)

S5 Fig Confirmation that exfoliative primary tumors are consistent for cHSA. Immunohistochemical evaluation of primary tumors from Dogs 1–3 and 5, confirming cHSA diagnosis based upon H&E and CD31 immunoreactivity.
(TIF)
