# Supplementary figures and images for: Overexpression of prostate specific membrane antigen by canine hemangiosarcoma cells provides opportunity for the molecular detection of disease burdens within hemorrhagic body cavity effusions
Source: PLoS One. 2019 Jan 2;14(1):e0210297. doi: 10.1371/journal.pone.0210297 (PMC6314605; doi:10.1371/journal.pone.0210297)

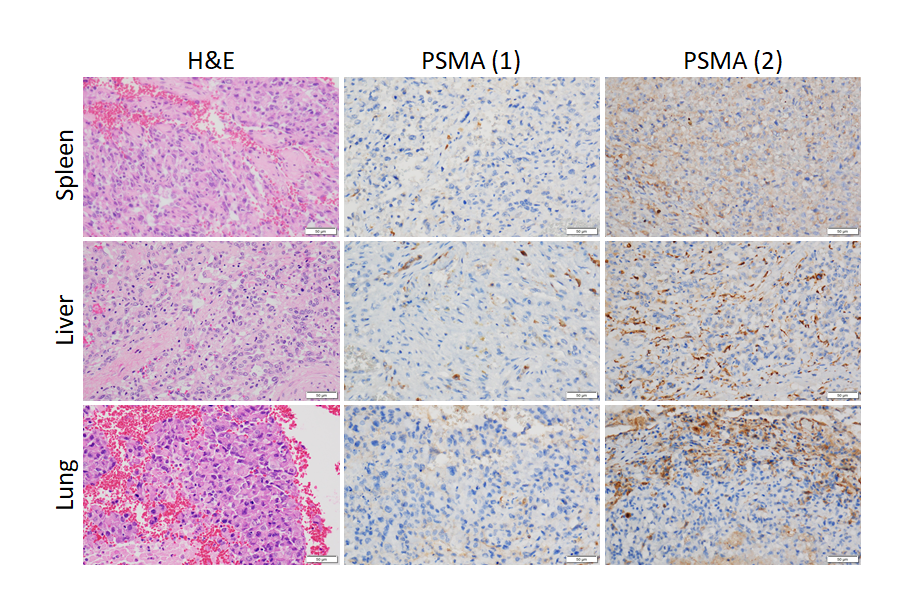

Supplement: S1 Fig — Representative H&E and PSMA immunoreactivity (scores 1 and 2) of selected cHSA tissue samples involving spleen, liver, and lung parenchyma. (TIF) [file pone.0210297.s001.tif]

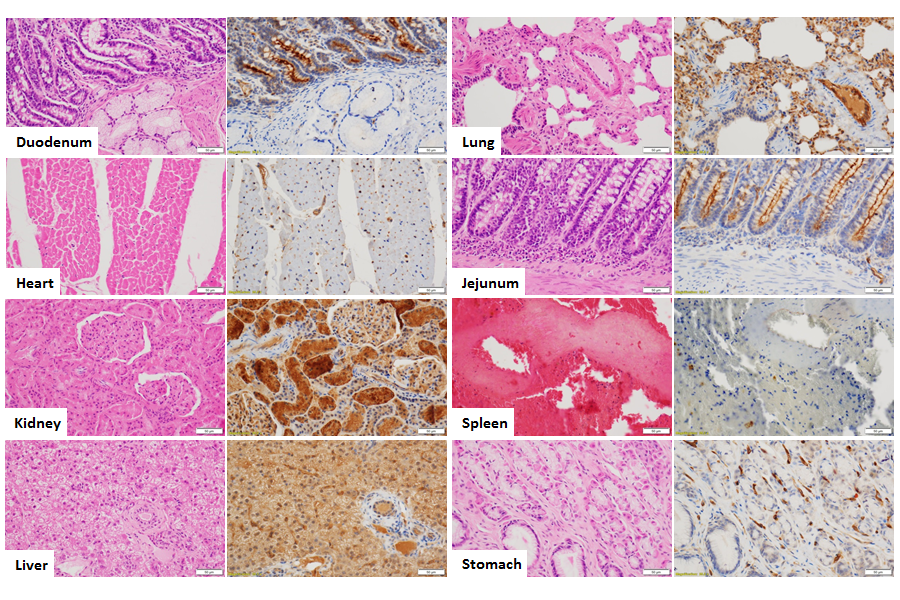

Supplement: S2 Fig — Representative H&E and PSMA immunoreactivity of select canine tissues derived from a commercial normal canine tissue microarray containing 28 cores. Confirmation of expected PSMA immunoreactivity based upon Human Protein Atlas data, with the exception of lung, which is strongly positive in canine only. (TIF) [file pone.0210297.s002.tif]

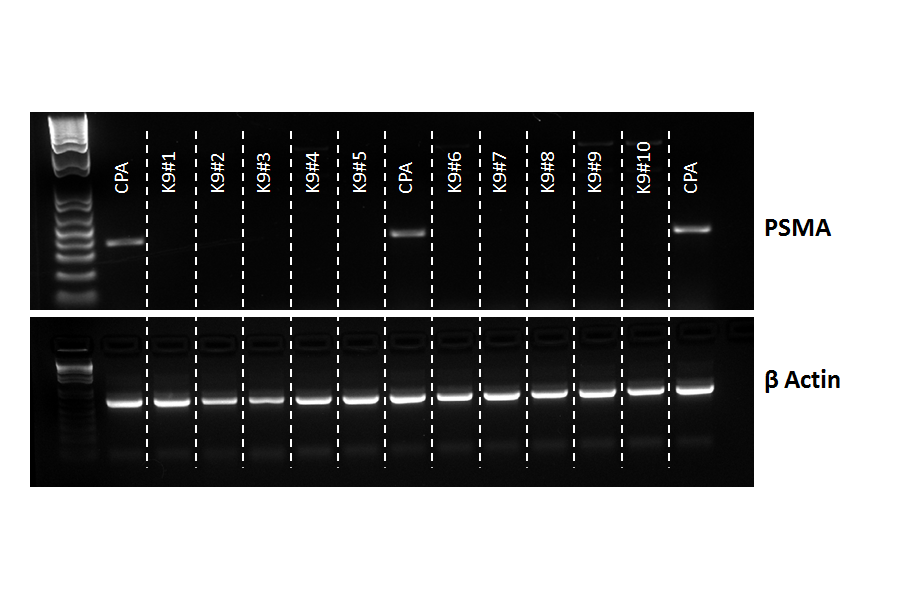

Supplement: S4 Fig — Evaluation of PSMA amplicon generation by qualitative PCR methodology (27 cycles) using 2 mL of EDTA whole blood collected from 10 healthy, young pet dogs (K9#1–10). No visible amplicons produced from whole blood of healthy dogs. CPA serves as positive PSMA control in lanes 1, 7, and 13. (TIF) [file pone.0210297.s004.tif]

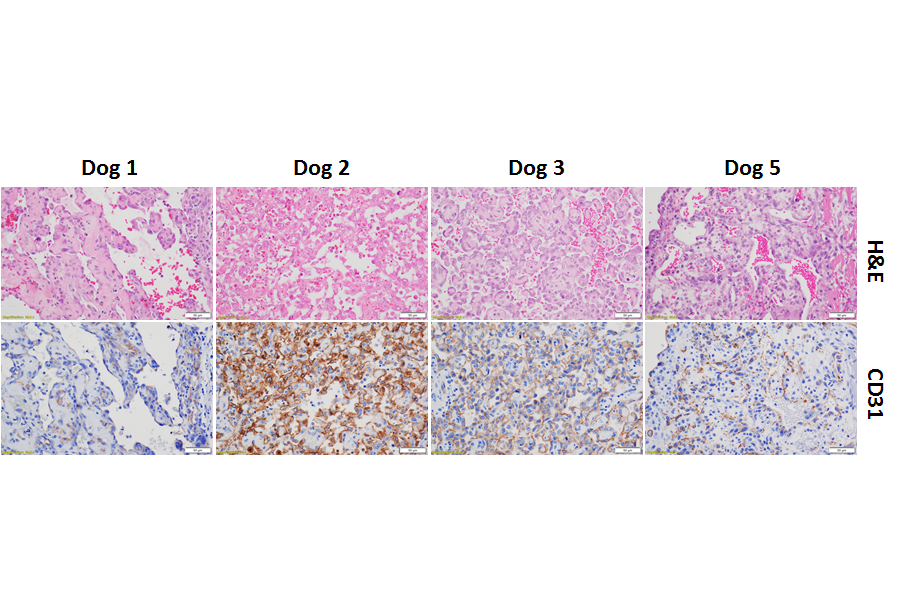

Supplement: S5 Fig — Immunohistochemical evaluation of primary tumors from Dogs 1–3 and 5, confirming cHSA diagnosis based upon H&E and CD31 immunoreactivity. (TIF) [file pone.0210297.s005.tif]
